# Assessing Disease Activity in Pediatric Crohn’s Disease Using Ultrasound: The Pediatric Crohn Disease Intestinal Ultrasound Score

**DOI:** 10.1097/MPG.0000000000003727

**Published:** 2023-02-07

**Authors:** Elsa A. van Wassenaer, Rick R. van Rijn, Floris A.E. de Voogd, Joost van Schuppen, Angelika Kindermann, Tim G.J. de Meij, Johan E. van Limbergen, K.B. Gecse, Geert R. D’Haens, Marc A. Benninga, Bart G.P. Koot

**Affiliations:** From the * Pediatric Gastroenterology, Emma Children’s Hospital, Amsterdam UMC, University of Amsterdam, Amsterdam, The Netherlands; the † Amsterdam Reproduction & Development Research Institute, Amsterdam UMC, University of Amsterdam, Amsterdam, The Netherlands; the ‡ Amsterdam Gastroenterology Endocrinology Metabolism Research Institute, Amsterdam UMC, University of Amsterdam, Amsterdam, The Netherlands; the § Radiology and Nuclear Medicine, Amsterdam UMC, University of Amsterdam, Amsterdam, The Netherlands; the ‖ Gastroenterology and Hepatology, Amsterdam UMC, University of Amsterdam, Amsterdam, The Netherlands; the ¶ Pediatric Gastroenterology Amsterdam, Emma Children’s Hospital, Amsterdam UMC, Vrije Universiteit Amsterdam, Amsterdam, The Netherlands.

**Keywords:** disease monitoring, intestinal ultrasound, pediatric Crohn disease

## Abstract

Currently, there is no consensus on how to score Crohn disease (CD) activity assessed by intestinal ultrasound (IUS) in children. This study aimed to design an easy-to-use IUS score for disease activity in pediatric CD.

**Methods:**

Children undergoing ileo-colonoscopy for CD assessment underwent IUS the day before ileo-colonoscopy, assessed with simple endoscopic score for CD (SES-CD). IUS features were compared to the SES-CD on segmental level. Multiple regression analyses, separately for terminal ileum (TI) and colon, were done to assess predictors of disease activity and to develop a model.

**Results:**

In 74 CD patients (median 15 years, 48% female), 67 TI and 364 colon segments were assessed. Based on receiver operating characteristics curves, bowel wall thickness (BWT) was categorized into low [1 point: 2–3 mm (TI) and 1.6–2 mm (colon)], medium [2 points: 3.0–3.7 mm (TI) and 2.0–2.7 mm (colon)], and high [3 points: >3.7 mm (TI) and >2.7 mm (colon)]. In TI, only BWT was retained in the model [high BWT: odds ratio (OR) 11.50, *P* < 0.001]. In colon, BWT (high BWT: OR 8.63, *P* < 0.001) and mesenteric fat (1 point: OR 3.02, *P* < 0.001) were independent predictors. A pediatric Crohn disease IUS score (PCD-US) cut-off of 1 resulted in a sensitivity of 82% (95% confidence interval, CI: 65%–93%) and 85% (95% CI: 80%–89%) and a cut-off of 3 in a specificity of 88% (72%–97%) and 92% (87%–96%) for TI and colon, respectively. Inter-observer agreement was moderate for TI and colon (*K*: 0.42, *K*: 0.49, respectively).

**Conclusions:**

The PCD-US score is an easy-to-use and reliable score to detect or rule out CD activity on segmental level in children. External validation is needed before applying this score in clinical practice.

**What Is Known**

- Transmural healing is increasingly recognized as treatment target for children with Crohn disease (CD).
- Transmural healing can be assessed with intestinal ultrasound (IUS).
- Studies in adults with CD show excellent accuracy for IUS.

**What Is New**

- This is the first prospective study to design an activity index for IUS in children with CD.
- The pediatric CD IUS score (PCD-US) is an easy-to-score index.
- The PCD-US can be used both to detect and to rule out CD activity.

Pediatric Crohn disease (CD) is a chronic debilitating condition, causing inflammation of the gastrointestinal tract (1). Due to its relapsing and remitting disease course, disease activity and preferably also disease location and extent should be monitored (2). Mucosal healing is one the most important treatment targets, as it predicts sustained clinical remission (3,4), and the importance of transmural healing is now also increasingly recognized (5,6). Biochemical markers such as C-reactive protein (CRP) and fecal calprotectin (FC) are often used to monitor disease activity, but these markers do not provide information on disease location and extent. Ileo-colonoscopy is considered as the gold standard diagnostic test, as mucosal healing and disease extent and location can accurately be assessed. However, this is an invasive procedure with risk of complications and high costs. Moreover, transmural healing cannot be assessed by ileo-colonoscopy. Magnetic resonance enterography (MRE) is a cross-sectional imaging modality that enables assessment of transmural healing (7). It is used in clinical practice for small bowel imaging especially at diagnosis, but its use in disease monitoring is limited by its costs and complexity due to need for oral and sometimes intravenous contrast agents, and need for patients to lay still. Moreover, the accuracy of MRE for assessing colonic lesions is poor (8). Intestinal ultrasound (IUS) is an alternative cross-sectional imaging modality. In the adult population, the accuracy for detecting active disease was excellent according to a meta-analysis (pooled sensitivity 88%, 95% confidence interval, CI: 85%–91% and pooled specificity 97%, 95% CI: 96%–98%) (9), both for small bowel and colon. However, as was demonstrated in a systematic review, there is currently no consensus on the definition of an abnormal IUS result in the pediatric population, and it is unclear whether small and large bowel should be scored the same. In addition, there is a lack of a standardized scoring system for use in the pediatric CD population. Only 2 indices for pediatric inflammatory bowel disease (IBD) have been proposed: 1 for pediatric ulcerative colitis (10) and 1 for both types of IBD based on retrospective data (11). Standardization of IUS scoring is needed before implementing IUS in clinical practice (12,13). Therefore, this prospective study aimed to develop an easy-to-use segmental score for assessing disease activity and severity in terminal ileum (TI) and colon in children with CD, that can be used during follow-up: the pediatric Crohn disease intestinal ultrasound score (PCD-US).

## MATERIALS AND METHODS

### Patient Population

In this prospective study, the RAINBOW study, consecutive children visiting the pediatric gastroenterology outpatient department of 2 tertiary care centers in Amsterdam were invited to participate between August 2019 and June 2021. Inclusion criteria were: 6–17 years of age, and undergoing an ileo-colonoscopy for diagnosis or follow-up of CD. Children were excluded in case of infectious gastro-enteritis, a histologically proven cytomegalo virus (CMV) infection, pregnancy, or a history of surgical resection of the intestine. In addition, children were excluded from the analysis if their final diagnosis was not CD according to the Porto criteria (14). Parts of the included subjects have been reported previously in a different analysis (15).

### Study Design

Participants underwent an IUS on the day before the ileo-colonoscopy, before start of the bowel preparation. In case this could not be realized, IUS was performed within 7 days before or after ileo-colonoscopy. The ileo-colonoscopy served as reference standard for the TI and colon. Baseline demographics, including age, gender, body mass index, Paris Classification, Pediatric Crohn Disease Activity Index (PCDAI), CRP, and FC were collected from the medical files.

### Intestinal Ultrasound

The IUS was performed by an ultrasonographer (EAW), who followed the International Bowel Ultrasound curriculum prior to the start of the study, and who had performed at least 100 IUS examinations under supervision. The ultrasonographer was unaware of the clinical details and was blinded for the results of the reference standard. Participants did not receive a specific bowel preparation, but were asked not to drink carbonated fluids or eat solid food 4 hours prior to the IUS, as is recommended by the ESPR-ESGAR guidelines (16). The IUS examinations were performed with a Philips EPIQ 5 machine (Philips Healthcare, Best, the Netherlands), using a convex probe (2–9 MHz, C9-2, Philips Healthcare, Best, the Netherlands) for general screening and a linear probe (4–12 MHz, L12-4, Philips Healthcare, Best, the Netherlands) for the bowel measurements. In order to assess inter-observer agreement, the IUS was performed a second time by a trained and experienced pediatric radiologist with >12 years of experience in a random subset of patients. The radiologist performed the IUS directly after the first examination, and was blinded for the previous results, as well as for clinical details and reference standard outcomes.

### IUS Measurements

Measurements were performed in TI, caecum, ascending-, transverse-, descending-, and sigmoid colon. The TI was identified near the psoas muscle and right iliac vessels, just proximal of the ileocecal valve, and was then followed to the caecum and ascending colon. The transverse colon was identified caudally from the stomach. The descending colon was identified on the lateral left side of the abdomen. The sigmoid colon was identified near the psoas muscle and left iliac vessels. The IUS measurements are displayed in the Supplemental Digital Content, http://links.lww.com/MPG/D73. We selected these items based on a study from IUS experts that used the Delphi method to identify the most important items of inflammation, and on 2 systematic reviews: 1 systematic review from 2019 evaluating all studies on diagnostic accuracy of IUS in children with IBD, and 1 systematic review summarizing all previously published IUS scoring indices for adult patients with IBD (Supplemental Digital Content, http://links.lww.com/MPG/D73) (12,13,17). The interrater variability of these items have been previously described (17–19).

The results of all measurements for each intestinal segment were recorded on a scoring form during the examinations.

### Ileo-Colonoscopy

Participants were prepared for the ileo-colonoscopy with Kleanprep or Moviprep (Norgine BV) according to local protocol. The ileo-colonoscopy, the reference standard for TI and colon, was performed by experienced pediatric gastroenterologists (all >10 years of experience), and video-recorded. Videos were centrally read and scored using the previously validated simple endoscopic score for CD (SES-CD) (20) per segment by 1 experienced pediatric gastroenterologist (BGPK, performed >300 ileo-colonoscopies before start of the study) who was trained to score the SES-CD score prior to the start of the study and who was blinded for the results of the IUS. As SES-CD is reported a total bowel score, the grading of SES-CD score (range 0–12 points) was transformed into a segmental bowel score as outlined below. This grading was based on expert opinion and designed in such a way that large ulcers would always be scored as moderate-severe disease, and aphthous ulcers scattered over a small (<10%) part of the segment as mild disease.

- No inflammation: 0 points
- Mild inflammation: 1–3 points
- Moderate-severe inflammation: >3 points

### Statistical Analyses and Development of the PCD-US

#### Prediction Model Building

Analyses were performed separately for the TI and for all colon segments collectively. This was based on the difference in bowel wall characteristics for these segments, particularly bowel wall thickness (BWT). In case either the IUS or the endoscopy data was missing, the segment was excluded from the analyses. For all segments we tested whether each IUS item in Table 1, Supplemental Digital Content, http://links.lww.com/MPG/D73 differed between the 3 endoscopic disease severity grades. Differences in continuous variables were assessed with Kruskal-Wallis test and differences in categorical variables with the *X*^2^ test. To simplify the interpretation of BWT measurements within the future model, BWT was subsequently categorized into 4 categories (normal, low, medium, high). As the rationale for cut-off values reported in previous pediatric studies is unclear, we based our cut-off values on receiver operating characteristics (ROC) curves. To assess the full range of diagnostic properties of BWT, we determined the cut-off associated with (1) around 10% false negatives, (2) optimal sensitivity and specificity, and (3) around 10% false positives.

Next we performed either multiple ordinal regression analysis or—if group sizes were too small—multiple logistic regression analysis, again separately for the TI, and all colon segments collectively, using the categorized reference standards as dependent variable. In case of logistic regression, we dichotomized the reference standard into no inflammation (ie, SES-CD = 0) versus inflammation (ie, SES-CD > 1). We first performed univariate analyses and for item reduction, we subsequently entered variables with the lowest *P* values and highest odds ratio (OR) 1 by 1 into the model (ie, stepwise forward selection). If the pseudo *R*^2^ (for ordinal regression) or −2 log-likelihood (for logistic regression) improved by 10% after entering a new variable, the variable was added to final model.

#### Weighing and Testing of Items

To further simplify our model into a score, we assigned points to the items in our final model, based on the rounded estimates or *B* values. To test the quality of this newly developed PCD-US score, we calculated sensitivity and specificity values and positive and negative likelihood ratios (LRs) for various cut-off values of the score, for differentiating active disease from remission, mild disease from remission, and moderate-severe disease from remission and mild disease.

#### Internal Validation and Reliability

We further validated our score internally, using bootstrapping with 1000 samples, and by assessing the correlation of the sum of the segmental PCD-US scores with the SES-CD total score, using Spearman rank correlation. For reliability, we calculated weighted kappa values to test inter-observer agreement of the PCD-US score. Kappa values were interpreted as follows: ≤0, no agreement; 0.01–0.20, none to slight; 0.21–0.40, fair; 0.41–0.60, moderate; 0.61–0.80, substantial; and 0.81–1.00, almost perfect agreement (21).

#### Sample Size

By including 74 participants, with an expected sensitivity of 85% and an expected specificity of 93%, based on a meta-analyses in the adult population (22), we hypothesized to be able to develop an accurate score, accepting a 95% confidence interval of 10% and expecting a prevalence of active disease among patients undergoing ileo-colonoscopy of 75%. This sample size would also allow to enter 3–5 variables in the model based on the rule of thumb of presence of minimally 25 inflamed segments and the number of variables being maximally the square root of the number of observations (23).

All analyses were done using SPSS v. 26. *P* values of <0.05 were considered significant.

### Ethical Considerations

Informed consent was obtained from all patients aged between 12 and 18 years and all caregivers of patients aged <16 years. This study was approved by the institutional review board of the Amsterdam UMC, location AMC.

## RESULTS

### Patient Population

**FIGURE 1.**
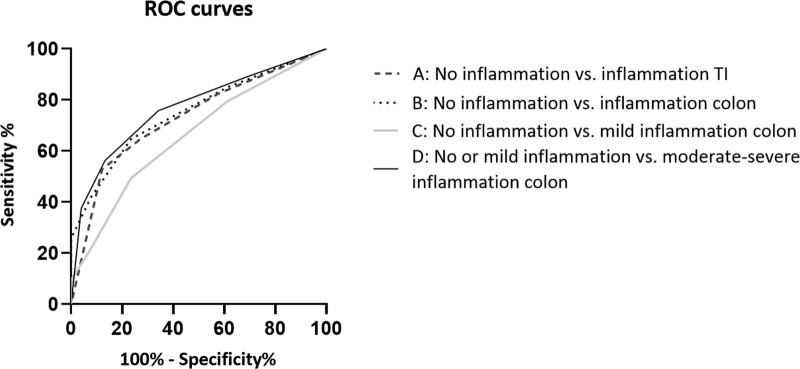
Receiver operating characteristics (ROC) curve for the PCD-US score. Area under the curves (95% CI) are A: 0.73 (0.61–0.85); B: 0.75 (0.70–0.80); C: 0.66 (0.59–0.73); and D: 0.77 (0.72–0.82). PCD-US = pediatric Crohn disease intestinal ultrasound score.

A total of 74 children were included [median age of 15 (interquartile range, IQR: 13–16) years] (Figure 1, Supplemental Digital Content, http://links.lww.com/MPG/D73). Baseline characteristics are displayed in Table 1. Most children (n = 56, 68%) were newly diagnosed with CD, which is also reflected by a high baseline PCDAI [27 (standard deviation [SD] 17)], FC (1538 mg/g, IQR: 503–3391), and SES-CD total score (11, IQR: 6–18). A total of 67 endoscopically assessed TI segments and 364 colon segments could be included; the ileum could not be scored in 7 children (in 1 the TI was not recorded on video and could thus not be scored, and in 6 the TI was not intubated; in 2 of these a stricture was seen on imaging, in 2 the ileocecal valve could not be identified, and in 2 the endoscope could not be introduced further), and the caecum and the sigmoid could not be assessed in 3 cases due to insufficient bowel cleanliness. These segments were left out of the analysis.

**TABLE 1.** Baseline characteristics

	N = 74		
Median [IQR] age	15 [13–16] y		
Gender	37 (50%) female		
Median [IQR] BMI	19 [16–21]		
Median [IQR] disease duration	0 [0–1] y		
Paris classification			
A1a – diagnosis before age of 10 y	9 (12%)		
A1b – diagnosis after age of 10 y	65 (88%)		
L1 – ileocecal disease	11 (15%)		
L2 – colonic disease	24 (32%)		
L3 – ileo-colonic disease	38 (51%)		
L4a – upper gastro-intestinal disease	37(50%)		
L4b – distal small bowel disease	9 (12%)		
B1– uncomplicated disease behavior	61 (82%)		
B2– stricturing disease behavior	9 (12%)		
B3– penetrating disease behavior	2 (3%)		
B2,3 – both B2 and B3	2 (3%)		
P– peri-anal disease	15 (20%)		
G1– growth delay	9 (12%)		
Laboratory values			
Median CRP	11 [2–33] mg/L		
Median [IQR] fecal calprotectin	1773 [549–3643] mg/g		
SES-CD total score	11 [6–18]		
Endoscopic activity	None	Mild	Moderate-severe
Terminal Ileum (n = 67)	32 (47%)	5(9%)	29 (43%)
Caecum (n = 71)	23 (32%)	18 (25%)	30 (42%)
Ascending colon (n = 74)	27 (36%)	14(19%)	33 (45%)
Transverse colon (n = 74)	32(43%)	19 (26%)	23 (31%)
Descending colon (n = 74)	27 (36%)	20 (27%)	27 (36%)
Sigmoid colon (n = 71)	27 (38%)	26 (37%)	18 (25%)

BMI = body mass index; CRP = C-reactive protein; IQR = interquartile range; SES-CD = simple endoscopic score for Crohn disease.

### Prediction Model Building

The IUS features per disease activity grade are displayed in Tables 2a and 2b. There was a significant difference in all IUS items between 3 disease activity grades for colon segments (*P* ≤ 0.007 for all items). For TI there was a significant difference in all IUS items except for presence of lymph nodes. Complications were noted by IUS in only 8 patients [stenosis in TI: n = 5, fistula in combination with abscess: n = 3 (2 in TI, 1 in sigmoid)].

The area under the receiver operating characteristic (AUROC) of BWT for detecting moderate-severe disease was 0.78 (95% CI: 0.67–0.90), and 0.78 (95% CI: 0.73–0.83) in TI and colon, respectively. The two cut-off values for BWT based on the ROC curve for each segment are displayed in Table 4a, Supplemental Digital Content, http://links.lww.com/MPG/D73).

### Multivariate Regression Analyses

For TI, the number of mildly inflamed segments was too low for ordinal regression; hence we performed logistic regression comparing no inflammation versus inflammation. Categorized BWT, Doppler signal, wall layer stratification, and peristalsis were predictors for endoscopic disease activity in univariate analyses (see Supplemental Digital Content, http://links.lww.com/MPG/D73). In the multivariate model, only BWT added to the predictive value of model for endoscopic TI disease activity (OR for high BWT: 11.50, *P* < 0.001) (Table 5a, Supplemental Digital Content, http://links.lww.com/MPG/D73).

For colon, all IUS items were independent predictors for endoscopic disease activity (see Table 4b, Supplemental Digital Content, http://links.lww.com/MPG/D73). The items that were retained in the ordinal multivariate model for optimal prediction of colonic endoscopic disease activity were BWT (OR for high BWT: 8.63, *P* < 0.001) and presence of mesenteric fat proliferation (OR 3.02, *P* < 0.001) (Table 5b, Supplemental Digital Content, http://links.lww.com/MPG/D73).

### Simplification Model into PCD-US Score

Based on the results of the multivariate regression analyses, a point-based index was made. The points assigned to each variable (ie, the calculation of the PCD-US score) are as follows:

**Table:** 

TI		Colon:	
BWT 2.0–3.0 mm:	1 point	BWT 1.6–2.0:	1 points
BWT 3.0–3.7 mm:	2 points	BWT 2.0–2.7 mm:	2 points
BWT >3.7 mm:	3 points	BWT >2.7 mm:	3 points
		Mesenteric fat infiltration:	1 point

Examples of IUS measurements are displayed in Figures 2 and 3, Supplemental Digital Content, http://links.lww.com/MPG/D73. The score was calculated for each segment. The ROC curves of the PCD-US score and their area under the curves (AUC) are displayed in Figure 1. Testing characteristics of the PCD-US scores are displayed in Tables 3a and 3b. A score of 3 points had a high probability of disease activity in both TI (specificity 88%) and colon (specificity 92%). In our cohort 29 out of 33 TI segments (88%), and 122 out of 133 colon segments (92%) could be classified correctly as not inflamed. A negative score (0 points) had a high probability of no disease activity (sensitivity 82% and 85% for TI and colon, respectively). In the study population, 28 out of 34 TI segments (88%), and 188 out of 221 (85%) colon segments could be classified correctly as inflamed. A total of 35% of TI segments and 42% of colon segments scored 1 or 2 points and could thus not be classified with sufficient accuracy. In the 7 patients where the ileum could not be intubated, the median PCD-US score was 3 (range 1–3).

### Internal Validation

The 95% confidence interval of the estimates and *B* values resulting from the bootstrapping are displayed in Tables 5a and 5b, Supplemental Digital Content, http://links.lww.com/MPG/D73. The PCD-US total score correlated significantly with the SES-CD total score (Spearman rho: 0.59, *P* < 0.01).

### Inter-Observer Agreement

A total of 251 segments (43 TI and 208 colon) were evaluated by both the ultrasonographer and the radiologist. The inter-observer agreement was moderate for TI (*K*: 0.42, SD: 0.13) and for colon (*K*: 0.49, standard error [SE]: 0.06). For TI, the first rater scored a PCD-US of 3 points in 3 of 43 segments while the second rater scored 0 points, and inversely, the second rater scored a PCD-US of 3 points in 1 of 43 segment where the first rater scored 0 points. For colon, the first rater scored a PCD-US of 3 points in 10 of 208 segments while the second rater scored 0 points, and inversely, the second rater scored a PCD-US of 3 points in 4 of 208 segments where the first rater scored 0 points.

## DISCUSSION

In this study, we developed and internally validated an easy-to-use activity index for IUS in children with CD—the PCD-US—consisting of 1 respectively 2 parameters, for the TI and for colon. In our cohort, the majority of segments could be classified correctly, using a cut-off value of ≥3 to detect disease activity (specificity 88% and 92% for TI and colon, respectively) and a cut-off of <1 to rule out disease activity (sensitivity 82% and 85% for TI and colon, respectively).

IUS is increasingly used and propagated as noninvasive and relatively cheap monitoring tool for children with CD (2), and an increasing number of studies have demonstrated the predictive value of transmural healing as assessed by IUS in adults (24). However, studies of good methodological quality on the accuracy of IUS in children are lacking (12). This is the first IUS score that has been prospectively designed in a pediatric CD cohort. Other scores in the pediatric population were either designed for ulcerative colitis patients (10), or for both types of IBD and based on retrospective data (11). In addition, the rationale for selection of IUS items and cut-off values is not specified in most previous studies. In the adult population, several activity scores for CD have been designed as well, most of which include BWT as one of, or the only variable, and with cut-offs ranging between 3 and 5 mm (13,25). Sensitivity of these scores ranged from 68% to 98% and specificity from 40% to 100%. Thus far, there was no consensus in the literature on the optimal cut-off value for BWT in the pediatric CD population, with reported cut-off values ranging between 1.5 and 4 mm, where 3 mm was the most used. In addition, it was unclear whether TI and colon should be scored differently (12). Our results confirm that TI and colon inflammation are characterized by different IUS features; BWT was higher in TI, and other features of inflammation, such as hyperemia and mesenteric fat proliferation were also present more frequently in TI. The reason for this difference might be the higher concentration of Peyer patches in the TI, especially in younger patients (26).

Interestingly, when included in multivariate analyses together with BWT, other IUS items did not improve the predictive value of the model except for mesenteric fat proliferation for colon, despite their predictive value for endoscopic disease activity in univariate analyses. This finding contrasts to other studies, describing for instance the presence of Doppler signal or abnormal wall layer stratification as independent predictors (13), but confirms the European Federation of Societies for Ultrasound in Medicine and Biology (EFSUMB) statement that BWT is the most important IUS parameter for CD (27). This was also confirmed by a group of IUS experts that used the Delphi method to identify the key parameters of inflammation (17).

In our cohort, a PCD-US score of ≥3 points, that is, colonic BWT > 2.0 mm combined with fat infiltration, or a BWT > 3.7 or >2.7 mm for TI and colon respectively, had a high probability of endoscopic disease activity (specificity 88% and 92%, positive LR: 4.37 and 5.50 for TI and colon, respectively). If this high accuracy is confirmed following external validation, particularly for reassessment of disease during treatment, the PCD-US score could be applied as point-of-care test to predict endoscopically active disease in daily clinical practice. A negative score (0 points) demonstrated a low probability of active endoscopic disease (sensitivity 82% and 85%, negative LR: 0.42 and 0.39 for TI and colon, respectively). Specificity values of the PCD-US score were low for PCD-US scores of 1 and 2 points, implying that when active disease is suspected in these cases, these PCD-US scores are not sufficient to demonstrate disease activity. In our cohort, only one third of segments scored 1 or 2 points. Combination with other inflammatory markers, such as FC, might improve the accuracy in these cases. The effectiveness of this approach is supported by the results of Bots et al (19), who demonstrated increased specificity (93% vs 67%) when combining mildly increased BWT with raised FC in adult ulcerative colitis patients.

A remarkable finding of our study was the moderate inter-observer agreement, which stresses the importance of standardized measurements (17). Operator dependency is one of the most frequently mentioned downsides of IUS. The inter-observer variability of IUS has been studied before, although most previous studies based their analysis on the assessment of saved images, which positively affects the level of agreement. In our study, the second rater performed the IUS independently, and our results are in line with one other study on real-time inter-observer agreement (28). Disagreement where a segment was categorized as non-inflamed (PCD-US 0 points) versus inflamed (PCD-US ≥3 points) was a rare finding in our study. Therefore, the clinical relevance of the operator dependency seems limited in a setting with standardized measurements and well trained operators.

Strengths of this study are the prospective design, the short time period between IUS and reference standard, blinding procedures, central reading procedure of the reference standards, and adequate sample size, which is reflected by the small 95% confidence interval of the sensitivity and specificity, especially for the colon. There are, however, some limitations to this study as well. Ileo-colonoscopy and IUS are 2 completely different imaging methods and transmural healing cannot be assessed by ileo-colonoscopy, while all features of IUS, especially mesenteric fat proliferation, are indicators of transmural disease activity. This may have led to a number of incorrect false positives, as transmural activity can be present even in segments with a normal mucosa (29), and thus underestimation of the diagnostic accuracy of IUS. Secondly, most children in our cohort were newly diagnosed CD patients, which may have biased our results. We solved this by analyzing our results per segment, and thus including an evenly spread number of segments with no-, mild-, and moderate-severe disease activity. In addition, according to the Prediction model study Risk Of Bias Assessment Tool (30) predictors of a model should ideally not be based on univariate analyses, but rather on existing knowledge. In the current study, we based the IUS measurements on existing knowledge, but in the process of building the final model, we did base our selection on univariate analyses, as the rationale for selecting the most important predictors was not clear from previous research. This may have biased our outcomes, and this underscores the need for external validation. Another limitation is the lack of information on intra-observer agreement. To the best of our knowledge, there are no studies investigating this topic, as due to the nature of disease, performing a second IUS after a memory-wise reasonable time would result in different observations. For this reason, we did not perform this analyses either.

Future research should focus on the external validation of the PCD-US score in an independent cohort. In addition, as IUS is increasingly used as a tool to monitor response to treatment, the sensitivity to change the PCD-US scores in relation to response to treatment needs to be assessed in a longitudinal cohort. The clinical added value of the PCD-US score, when using it in combination with other inflammatory markers and its predictive value for a flare is currently being studied.

## CONCLUSIONS

In conclusion, we developed the PCD-US score, an easy-to-use IUS score, which reflects moderate-to-severe CD activity on a segmental level. Further validation studies are warranted to assess the performance of this score during follow-up of CD and its wider application in clinical practice.

**TABLE 2a.** IUS findings in TI per disease activity grade (based on ileo-colonoscopy)

	No inflammation, n=33	Mild inflammation, n= 5	Moderate-severe inflammation, n=29	*P* value
BWT [IQR] mm	2.22 [1.61–3.02]	1.74 [1.32–3.33]	4.22 [2.80–5.77]	<0.001
Doppler				0.006
No	14 (42%)	4 (80%)	6 (21%)	
Spots	12 (36%)	0 (0%)	5 (17%)	
Stretches	2 (6%)	1 (20%)	3 (10%)	
Into mesentery	5 (15%)	0 (0%)	15 (52%)	
Mesenteric fat proliferation	13 (39%)	0 (0%)	21 (72%)	0.002
Wall layer stratification	30 (91%)	3 (75%)	16 (55%)	0.006
Colonic haustrations	–	–	–	
Peristalsis visibility	28 (85%)	3 (60%)	12 (41%)	0.002
Lymph nodes	10 (30%)	1 (20%)	13 (45%)	NS

BWT = bowel wall thickness; IQR = interquartile range; IUS = intestinal ultrasound; TI = terminal ileum.

**TABLE 2b.** IUS findings in colon per disease activity grade (based on ileo-colonoscopy)

	No inflammation, n= 134	Mild inflammation, n= 96	Moderate-severe inflammation, n=131	*P* value
BWT [IQR] mm	1.67 [1.44–1.97]	1.91 [1.66–2.47]	2.76 [1.94–3.65]	<0.001
Doppler				<0.001
No	103 (79%)	58 (60%)	62 (47%)	
Spots	20 (15%)	31 (32%)	30 (23%)	
Stretches	5 (4%)	2 (2%)	16 (12%)	
Into mesentery	3 (2%)	5 (5%)	21 (16%)	
Mesenteric fat proliferation	7 (5%)	14 (15%)	58 (44%)	<0.001
Wall layer stratification	132 (92%)	86 (90%)	99 (76%)	0.007
Colonic haustrations	127 (95%)	89 (93%)	95 (73%)	<0.001
Peristalsis visibility	-	-	-	0.002
Lymph nodes	3 (2%)	11 (11%)	30 (23%)	<0.001

BWT: bowel wall thickness, IUS: intestinal ultrasound, IQR: interquartile range.

**TABLE 3a.** Testing characteristics of PCD-US score for endoscopic disease activity in TI

	Sensitivity (95% CI)	Specificity (95% CI)	LR+ (95% CI)	LR− (95% CI)
*No inflammation versus inflammation*				
≥1 point	82 (65–93)%	42 (25–61%)	1.43 (1.03–1.99)	0.42 (0.18–0.95)
≥2 points	65 (47–80)%	73 (54–87)%	2.37 (1.29–4.37)	0.49 (0.29–0.80)
3 points	53 (35–70)%	88 (72–97)%	4.37 (1.65–11.54)	0.54 (0.37–0.78)
*No or mild inflammation versus moderate-severe inflammation: –*				

CI = confidence interval; LR− = negative likelihood ratio; LR+ = positive likelihood ratio; PCD-US = pediatric Crohn disease ultrasound score; TI = terminal ileum.

**TABLE 3b.** Testing characteristics of PCD-US score for endoscopic disease activity in colon

	Sensitivity (95% CI)	Specificity (95% CI)	LR+ (95% CI)	LR− (95% CI)
*No inflammation versus inflammation*				
≥1 point	85 (80–89)%	39 (30–48)%	1.39 (1.20–1.60)	0.39 (0.26–0.57)
≥2 points	65 (58–71)%	77 (68–83)%	2.76 (1.99–3.81)	0.46 (0.38–0.56)
≥3 points	42 (36–48)%	92 (87–96)%	5.50 (2.97–10.17)	0.63 (0.56–0.71)
4 points	26 (20–32)%	100 (97–100)%	–	0.74 (0.69–0.80)
*No inflammation versus mild inflammation*				
≥1 point	80 (70–87)%	39 (30–48)%	1.30 (1.09–1.54)	0.53 (0.34–0.83)
≥2 points	49 (39–68)%	77 (68–83)%	2.11(1.45–3.05)	0.66 (0.53–0.82)
≥3 points	14 (9–21)%	88 (79–94)%	1.17 (0.58–2.38)	0.98 (0.88–1.08)
4 points	10 (5–18)%	100 (97–100)%	–	0.90 (0.85–0.97)
*No or mild inflammation versus moderate-severe inflammation*				
≥1 point	89 (82–94)%	31 (25–38)%	1.29 (1.16–1.44)	0.35 (0.21–0.60)
≥2 points	76 (67–83)%	66 (59–72)%	2.21 (1.80–2.72)	0.37 (0.27–0.51)
≥3 points	56 (47–59)%	87 (82–91)%	4.22 (2.92–6.09)	0.50 (0.41–0.62)
4 points	38 (29–46)%	96 (93–98)%	9.37 (4.76–18.47)	0.65 (0.57–0.75)

–: could not be calculated.

CI = confidence interval; LR− = negative likelihood ratio; LR+ = positive likelihood ratio; PCD-US: pediatric Crohn disease ultrasound score.

## Acknowledgment

We thank Hanneke van de Lee from the clinical research department of the Emma Children’s hospital for her contribution for the conception and design of this study.

## Supplementary Material


