# Supplementary material for: Assessing Disease Activity in Pediatric Crohn’s Disease Using Ultrasound: The Pediatric Crohn Disease Intestinal Ultrasound Score
Source: J Pediatr Gastroenterol Nutr. 2023 Feb 7;76(5):582–9. doi: 10.1097/MPG.0000000000003727 (PMC10097488; doi:10.1097/MPG.0000000000003727)
Supplement: Supplementary file 1 [file mpg-76-582-s001.pdf]

Supplementary file:

Suppl. Table 1: IUS items used in other studies		
**SR children (n=14)** (van Wassenaer et al., 2019)	**SR indices adults (n=25)** (Goodsall et al., 2021)	**Delphi study adults** (Novak et al., 2020)
BWT n= 13/14 (cut-offs: 1.5, 2, 2.5, 3, 4 mm)	BWT n= 25/26 (cut-offs: 3, 4, 5 mm)	BWT
Doppler n=9/14	Doppler n=15/25	WLS
Lymph nodes n= 6/14	Lymph nodes n=1/25	Doppler
Mesenteric fat n= 4/14	Mesenteric fat n=1/25	Mesenteric fat
WLS n=4/14	WLS= 15/25	
Colonic haustrations n=1/14	Colonic haustrations n=1	
Peristalsis n=0	Peristalsis n=1	
BWT: bowel wall thickness, IUS: intestinal ultrasound, SR: systematic review, WLS: wall layer stratification.		

Suppl. Table 2: IUS measurements	
Bowel wall thickness	Measured from the lumen/mucosa interface to the muscularis/serosa interface in a non-contracted bowel loop, next to a haustration at the most severely inflamed part of every bowel segment. BWT was measured twice in the longitudinal plane and twice in the cross-sectional plane and the mean of these four measurements was used.
Bowel wall perfusion	Colour Doppler (no /spots/ stretches/ stretches into mesentery)
Mesenteric fat proliferation	Yes or no
Visibility of colonic haustrations	Yes or no
Visibility of wall layer stratification	Yes or no
Peristalsis	Only for small bowel segments. Visible within 10 seconds? Yes or no
Presence of lymph nodes and size	Yes or no. Size measured at the short axis
Presence of complications	Abscesses, strictures, fistulas: yes or no

**Suppl. Table 3: cut-off values for bowel wall thickness (BWT) based on ROC-curves *online only***		
	**Sensitivity**	**Specificity**
Cut-off 1 : low BWT		
TI: BWT> 2.0 mm	90%	45%
Colon: BWT >1.6 mm	89%	31%
Small bowel: BWT> 1.7 mm	91%	41%
Cut-off 2 : medium BWT		
TI: BWT >3.0 mm	73%	76%
Colon: BWT> 2.0 mm	72%	69%
Small bowel: BWT> 2.1 mm	73%	80%
Cut-off 3 : high BWT		
TI: BWT>3.7 mm	62%	89%

Colon: BWT> 2.7 mm<br>Small bowel BWT> 2.5 mm	51%<br>46%	89%<br>90%
Cut-off associated with 1) around 10% false negatives, 2) optimal sensitivity and specificity, and 3) around 10% false positives.		

**Suppl. Table 4a: univariate logistic regression with IUS items for TI**				
**IUS item**	**B (SE)**	**OR**	**95%CI**	**p-value**
BWT 2.0-3.0 mm	0.34 (0.27)	1.41	0.35-5.64	0.20
BWT 3.0-3.7 mm	1.29 (0.29)	3.65	0.37-9.49	<0.001
BWT >3.7 mm	2.44 (0.38)	11.50	2.48-44.55	<0.001
Mesenteric fat infiltration	0.91 (0.50)	2.49	0.93-6.64	0.069
Doppler<br>Spots<br>Stretches<br>Stretches into mesentery	 -0.54 (0.67)<br>1.03 (0.96)<br>1.44 (0.66)	 0.58<br>2.80<br>4.20	 0.16-2.19<br>0.43-18.38<br>1.15-15.37	 0.42<br>0.28<br>0.03
Peristalsis	1.96 (0.60)	7.09	2.21-22.81	0.001
WLS-Abnormal wall layer stratification	2.0 (0.70)	7.37	1.87-29.08	0.004
BWT: bowel wall thickness, IUS: intestinal ultrasound, TI: terminal ileum, SE: standard error.				

**Suppl. Table 4b: univariate ordinal regression with IUS items for colon segments**				
**IUS item**	**Estimate (SE)**	**OR**	**95%CI**	**p-value**
BWT 1.6-2.0	0.29 (0.29)	1.34	0.77-2.35	0.30
BWT 2.0-2.7 mm	1.26 (0.30)	3.50	1.95-6.35	<0.001
BWT >2.7 mm	2.77 (0.33)	15.91	8.30-30.48	<0.001
Mesenteric fat infiltration	2.09 (0.28)	8.10	4.65-14.08	<0.001
Doppler<br>Spots<br>Stretches<br>Stretches into mesentery	 0.66 (0.24)<br>1.69 (0.39)<br>1.98 (0.34)	 1.95<br>5.42<br>7.24	 1.21-3.12<br>2.23-13.17<br>3.10-16.89	 0.006<br><0.001<br><0.001
Abnormal colonic haustrations	1.68	5.35	2.81-10.21	<0.001
WLS-Abnormal wall layer stratification	1.84 (0.36)	6.32	3.11-12.82	<0.001
Lymph nodes	1.64 (0.34)	5.15	2.63-10.09	<0.001

Estimate = predicted change in log odds for being in higher SES-CD category, corrected for other variables in model. BWT: bowel wall thickness, IUS: intestinal ultrasound, SE: standard error.

**Suppl. Table 5a: IUS items in final model for TI segments**					
**IUS item**	**B (SE)**	**95% CI***	**OR**	**95%CI**	**p-value**
BWT 2.0-3.0 mm	0.34 (0.27)	-0.19-0.88	1.41	0.35-5.64	0.20
BWT 3.0-3.7 mm	1.29 (0.29)	0.72-1.90	3.65	0.37-9.49	<0.001
BWT >3.7 mm	2.44 (0.38)	1.74-3.28	11.50	2.48-44.55	<0.001
Pseudo R^2: 0.15 (Cox&Snell), 0.20 (Nagelkerke).<br>BWT: bowel wall thickness, IUS: intestinal ultrasound, TI: terminal ileum<br>* Result from bootstrapping					

**Suppl. Table 5b: IUS items in final model for colon segments**					
**IUS item**	**Estimate (SE)**	**95% CI***	**OR**	**95%CI**	**p-value**
BWT 1.6-2.0	0.35 (0.29)	-0.31-0.94	1.28	0.73-2.25	0.39
BWT 2.0-2.7 mm	1.34 (0.30)	0.52-1.80	3.13	1.72-5.67	<0.001
BWT >2.7 mm	2.16 (0.37)	1.48-3.00	8.63	4.20-17.76	<0.001
Mesenteric fat infiltration	1.10 (0.33)	0.42-1.88	3.02	1.58-5.76	<0.001
Estimate = predicted change in log odds for being in higher SES-CD category, corrected for other variables in model.<br>Model fitting information: -2Log Likelihood p-value <0.001, goodness of fit p-value >0.05, Pseudo R^2: 0.27 (Cox&Snell), 0.30 (Nagelkerke). Test of parallel lines p-value >0.05.<br>BWT: bowel wall thickness, IUS: intestinal ultrasound<br>* Result from bootstrapping					

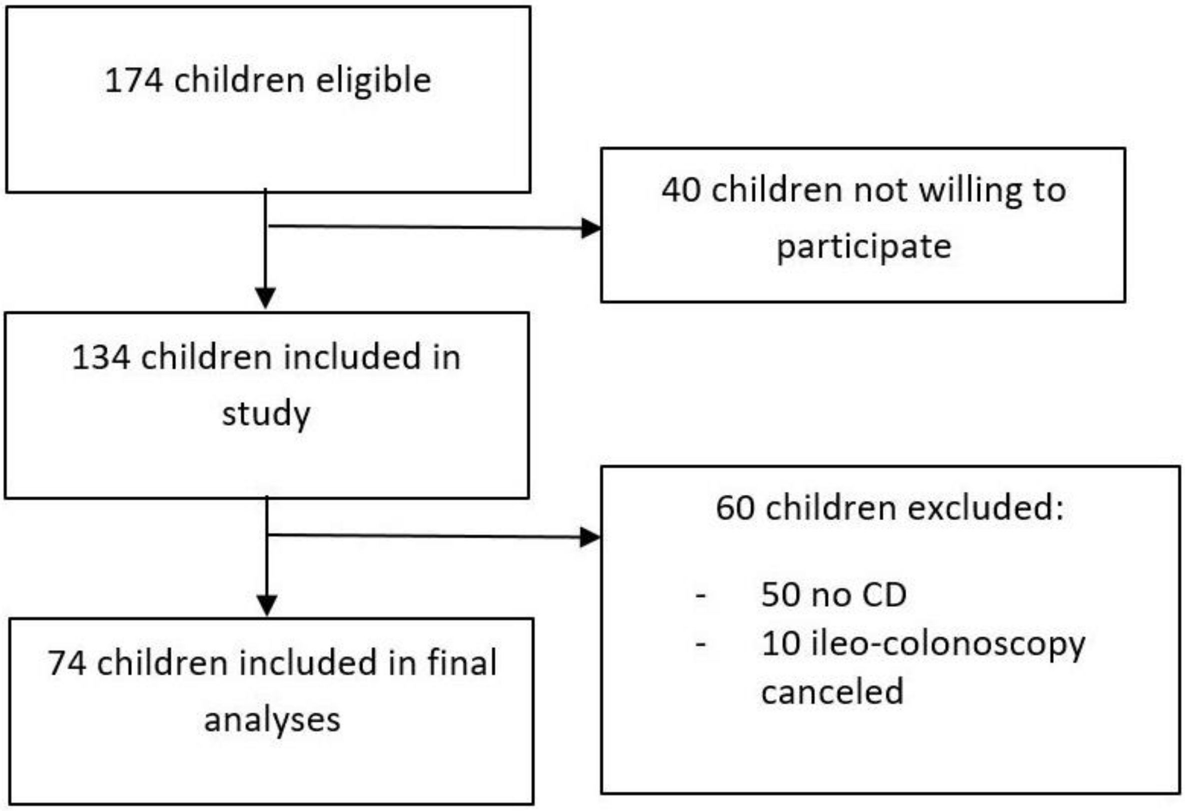

Suppl. Figure 1 Flowchart of patient selection. CD: Crohn's Disease

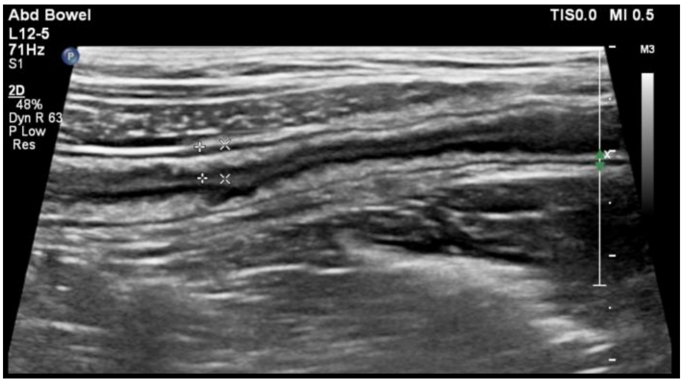

Suppl. Figure 2: longitudinal image of bowel segment, with bowel wall thickening

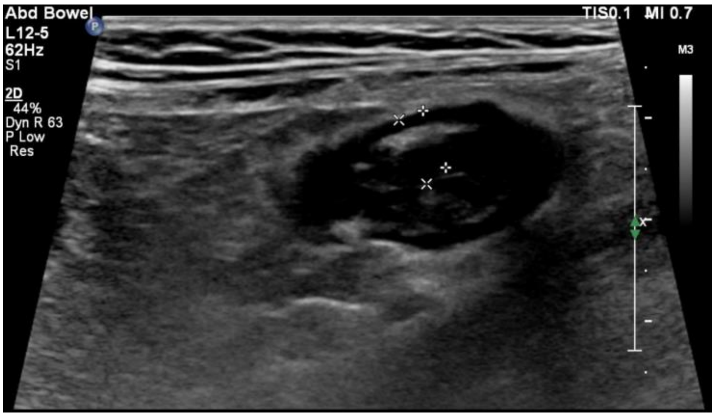

Suppl. Figure 3: cross-sectional image of bowel segment, with bowel wall thickening and surrounding mesenteric fat proliferation.
